# The expression pattern of matrix-producing tumor stroma is of prognostic importance in breast cancer

**DOI:** 10.1186/s12885-016-2864-2

**Published:** 2016-11-04

**Authors:** Sofia Winslow, Kajsa Ericson Lindquist, Anders Edsjö, Christer Larsson

**Affiliations:** 1 Department of Laboratory Medicine, Lund University Cancer Center, Translational Cancer Research, Lund University, Lund, Sweden; 2 Department of Pathology, Regional Laboratories Region Skåne, Lund, Sweden; 3 Department of Clinical Sciences Lund, Division of Oncology and Pathology, Lund University, Lund, Sweden; 4 Department of Pathology, Sahlgrenska Cancer Center, Institute of Biomedicine, Sahlgrenska Academy, University of Gothenburg, Gothenburg, Sweden; 5 Institute of Biochemistry I, Faculty of Medicine, Goethe-University Frankfurt, Frankfurt, Germany

**Keywords:** Breast cancer, Tumor stroma, ECM metagenes, TCF4, Endothelial metagenes, P4HA3

## Abstract

**Background:**

There are several indications that the composition of the tumor stroma can contribute to the malignancy of a tumor. Here we utilized expression data sets to identify metagenes that may serve as surrogate marker for the extent of matrix production and vascularization of a tumor and to characterize prognostic molecular components of the stroma.

**Methods:**

TCGA data sets from six cancer forms, two breast cancer microarray sets and one mRNA data set of xenografted tumors were downloaded. Using the mean correlation as distance measure compact clusters with genes representing extracellular matrix production (ECM metagene) and vascularization (endothelial metagene) were defined. Explorative Cox modeling was used to identify prognostic stromal gene sets.

**Results:**

Clustering of stromal genes in six cancer data sets resulted in metagenes, each containing three genes, representing matrix production and vascularization. The ECM metagene was associated with poor prognosis in renal clear cell carcinoma and in lung adenocarcinoma but not in other cancers investigated. Explorative Cox modeling using gene pairs identified gene sets that in multivariate models were prognostic in breast cancer. This was validated in two microarray sets. Two notable genes are *TCF4* and *P4HA3* which were included in the sets associated with positive and negative prognosis, respectively. Data from laser-microdissected tumors, a xenografted tumor data set and from correlation analyses demonstrate the stroma specificity of the genes.

**Conclusions:**

It is possible to construct ECM and endothelial metagenes common for several cancer forms. The molecular composition of matrix-producing cells, rather than the extent of matrix production seem to be important for breast cancer prognosis.

**Electronic supplementary material:**

The online version of this article (doi:10.1186/s12885-016-2864-2) contains supplementary material, which is available to authorized users.

## Background

Along with malignant cells, tumors contain a complex microenvironment which consists of an extracellular matrix (ECM) and a large variety of non-cancerous stromal cells. The microenvironment is in constant interaction with the cancer cells and becomes modified during tumor progression, exemplified by vascularization, remodeled ECM and augmented tissue stiffness [1–3]. During remodeling, the ECM undergoes a desmoplastic reaction generating a fibrous tissue with many newly produced stromal proteins [4] which can further promote cancer progression [5, 6]. The ECM is composed of a variety of components, with fibroblast-produced collagens being one of the major proteins [7]. High expression of collagens have for instance been reported to associate with tumor metastasis in breast cancer [8] and women with collagen-rich dense breasts have an increased risk of developing breast cancer [9].

Stromal cells can also promote tumorigenesis by inducing an angiogenic switch which may contribute to a more aggressive phenotype of the tumor. This includes increased endothelial cell proliferation and microvessel density [10].

Global gene expression analyses have successfully been used to subgroup tumors and identify molecular characteristics that are of prognostic value. The most well-established example is perhaps the PAM50-based classification of breast cancers [11]. In many cases the profiles are based on gene expression presumably emanating from the cancer cells. However, there are also studies that have identified gene signatures based on stromal genes that have been indicated to predict clinical outcome in breast cancer [12–14] and other tumor forms [15, 16].

In a recent study we identified genes specific for or highly enriched in the stromal compartment of breast cancer tumors using global RNA analyses of laser-microdissected tumors followed by bioinformatics expansion by correlation analyses using The Cancer Genome Atlas (TCGA) breast cancer dataset [17]. When clustered, the genes could be subgrouped in several compact clusters representing either endothelial, immune response, or matrix-associated genes. None of the signatures were strongly associated with prognosis in univariate models. However, in a multivariate analysis two signatures were prognostic with opposite association with the hazard ratio, indicating that the molecular composition of an immune response is more important than the total extent of the response. This raises the question if a similar concept holds for matrix-related genes. Here we have tested the hypothesis that the molecular composition of the matrix gene expression profile of a tumor may be of prognostic importance.

## Methods

### Data sets

RNA-seq data for breast cancer, colon adenocarcinoma, kidney renal clear cell carcinoma, head and neck cancer, lung adenocarcinoma, lung squamous cell carcinoma, and normal breast tissue were downloaded from the TCGA data portal (Additional file 1: Table S1, [18]).

Breast cancer microarray data sets [19, 20] were downloaded from Array Express (Additional file 2: Table S2, [21] and RNA-seq data of human breast cancer cell lines grafted into mice were downloaded from GEO database (Accession: GSE66744) [22].

### Data analysis

All data analyses were performed with R. The TCGA data were log2-transformed after addition of 1 to each value. ECM and endothelial gene sets were expanded by selecting genes from the TCGA breast cancer data set that had a correlation coefficient above 0.84 with at least one gene in the seeding sets defined as genes in our previously defined signatures 1 and 2 (ECM) and in signature 4 and 5 (endothelial) [17]. To obtain compact gene clusters the correlation coefficients between all genes were calculated and the gene with the lowest mean of the correlation coefficients was removed from the set. This procedure was repeated until all the genes in the cluster had a mean correlation coefficient above 0.85. The aggregated value of the obtained ECM and endothelial metagenes for a tumor were calculated as the standardized mean of the log2 expression of all genes in the signature.

For explorative survival analyses the log2 expression of all genes in the expanded ECM set (Additional file 3: Table S3) were tested pairwise in a multivariate Cox proportional hazard model, stratified for ER and node status, using the TCGA breast cancer data. The pairs were ranked according to the *p*-value of the likelihood ratio test of the models. Genes appearing more than five times in the top 100 pairs were selected for inclusion in “poor” and “good” prognosis signatures. The survival package in R was used for all survival analyses. The R code and the signatures defined in [17] are included as Additional files 4, 5 and 6.

### Histological analysis of TCGA breast tumors

Histological images of TCGA breast tumors stained with hematoxylin and eosin were obtained from Cancer Digital Slide Archive [23]. Tumor stroma patterns were classified as “separated” or “mixed”. The stromal pattern was classified as “separated” when it was distinct and compactly organized surrounding a bulk tumorous structure whereas it was classified as “mixed” when the pattern was typified by disseminated stromal fibers mixed with the cancer cells (Additional file 7: Figure S1). The tumor was classified based on the dominating pattern. The amount of stroma in a tumor section was furthermore estimated as low, intermediate or high.

### Tumor material for laser microdissection

Formalin-fixed specimens of tumors that had been removed as part of standard care from patients that had given informed consent were obtained from Skåne University Hospital, Malmö, and stored at 4 °C until analysis. Ethical permission has been obtained from the local research ethics committee (Regionala etikprövningsnämnden i Lund, Dnr 2009/658). The tumors were negative for estrogen and progesterone receptors and had no ERBB2 (HER2) amplification according to the pathology reports. The tumors analyzed had been classified as grade II or grade III according to Nottingham histological grade. Three of the tumors were reported to be invasive ductal carcinoma, one ductal carcinoma in situ and one medullary carcinoma. Specimens with sufficient amount of stroma and stromal inflammation to enable RT-PCR analysis of laser-microdissected tumor compartments were selected.

### Tissue preparation, staining and laser microdissection

Sections of archived formalin-fixed paraffin-embedded breast tumor samples (5 μm) were mounted onto polyethylene terephthalate (PET) membrane slides (Leica Microsystems, Wetzlar, Germany) as described previously [17] and stained with cresyl violet LCM staining kit (Ambion, part of Thermo Fisher Scientific, Waltham, MA, USA) to optimize RNA quality. Tumor compartments were isolated with laser microdissection on a Leica LMD6500 and collected in AllPrep RNA/DNA FFPE kit lysis buffer (Qiagen, Hilden, Germany) with Proteinase K (Additional file 8: Figure S2).

### RNA extraction, reverse transcription and TaqMan RT-PCR

Total RNA was extracted and evaluated as described previously [17]. Quantive RT-PCR procedures were performed using reagents from Applied Biosystems, part of Thermo Fisher Scientific, Waltham, MA, USA. The High Capacity RNA-to-cDNA kit was used for reverse transcription and quantitative PCR was performed with TaqMan Gene expression master mix in QuantStudio 7 Flex Real-Time PCR system (2 min 50 °C, 10 min 95 °C, 40 cycles of 15 s 95 °C followed by 1 min 60 °C). Predesigned assays for the analyzed RNAs were obtained from the manufacturer (Additional file 9: Table S4). Expression levels were normalized to the expression of the reference genes *ACTB* and *UBC*.

## Results

### Gene signatures for ECM and endothelial tissue

An initial aim was to identify gene signatures that would indicate the amount of ECM-producing cells and endothelial density in a tumor. To achieve this we utilized the gene sets that we recently identified by global RNA analysis of laser-microdissected breast cancer tumors [17]. We used the genes in the two ECM-related signatures to expand the gene list by identifying all genes that in the TCGA breast cancer RNA-seq data had a correlation coefficient above 0.84 with at least one gene in the original sets (Additional file 3: Table S3). We thereby assume that we have gathered the genes that will have a conceivable potential as markers for the amount of ECM-producing cells such as fibroblasts. We thereafter reasoned that genes that are highly correlated and form a compact cluster may conceivably emanate from the same type of cells. Therefore, the gene list was narrowed down to a cluster defined as the genes for which the average of their correlation coefficients with other genes in the cluster was above 0.85.

Based on assumption that the tumor stroma may have common characteristics across cancer forms the process was reiterated for the TCGA colon adenocarcinoma, head and neck squamous cell carcinoma, kidney renal clear cell carcinoma, lung adenocarcinoma, and lung squamous cell carcinoma data sets. The resulting gene signatures for the cancer sets are shown in Table 1. Three genes were present in the signatures from all cancer sets, *COL1A1, COL1A2*, and *COL3A1.* These genes are considered to be expressed mainly in fibroblasts, and the fact they are highly correlated in all cancer forms suggest that the expression levels of the genes may represent fibroblast number in many different tumor types. These genes were therefore defined as the ECM metagene.

**Table 1:** ECM gene signatures in TCGA cancer sets

BRCA	COAD	COAD cont	HNSC	KIRC	LUAD	LUSC
*ADAM12*	*ADAM12*	*ITGA11*	*COL1A1*	*COL1A1*	*COL1A1*	*AEBP1*
*BNC2*	*AEBP1*	*LUM*	*COL1A2*	*COL1A2*	*COL1A2*	*COL1A1*
*CDH11*	*ANTXR1*	*MMP2*	*COL3A1*	*COL3A1*	*COL3A1*	*COL1A2*
*COL1A1*	*BNC2*	*MSRB3*	*COL6A1*	*COL5A1*	*COL5A1*	*COL3A1*
*COL1A2*	*C10orf72*	*OLFML1*	*COL6A3*	*COL5A2*	*COL5A2*	*COL5A1*
*COL3A1*	*CCDC80*	*OLFML2B*	*NID2*	*FAP*	*COL6A3*	*COL6A3*
*COL5A1*	*COL1A1*	*PCOLCE*	*OLFML2B*		*THBS2*	*NID2*
*COL5A2*	*COL1A2*	*PDGFRB*	*PDGFRB*			*PDGFRB*
*COL6A3*	*COL3A1*	*SPARC*	*POSTN*			*SPARC*
*DACT1*	*COL5A1*	*THBS2*	*SPARC*			*THBS2*
*FAP*	*COL5A2*	*THY1*	*TIMP2*			
*FBN1*	*COL6A2*	*TIMP2*	*VCAN*			
*GLT8D2*	*COL6A3*	*VCAN*				
*LUM*	*COL8A1*					
*POSTN*	*DCN*					
*SPARC*	*FAM26E*					
*THBS2*	*FBN1*					
*VCAN*	*FSTL1*					

The signatures were defined by an iterative process. Starting with the expanded ECM gene set (Additional file 3: Table S3) the gene with the lowest mean value of the correlation coefficients of the log2 expression with the genes in the set was removed from the set. The process was reiterated until all genes had a mean correlation coefficient above 0.85

We took the same approach with the endothelial gene sets. Also in this case three genes (*CDH5, CXorf36,* and *TIE1*) were present in the final cluster in all six cancer sets (Additional file 10: Table S5 and Table 2). These genes were therefore defined as the endothelial metagene.

**Table 2:** Endothelial gene signatures in TCGA cancer sets

BRCA	COAD	COAD cont	HNSC	KIRC	KIRC cont	LUAD	LUSC
*ARHGEF15*	*ARHGEF15*	*MMRN2*	*CD34*	*ARHGEF15*	*LDB2*	*ARHGEF15*	*CD93*
*CD34*	*BCL6B*	*MYCT1*	*CDH5*	*BCL6B*	*MMRN2*	*CD34*	*CDH5*
*CDH5*	*CALCRL*	*PCDH12*	*CXorf36*	*CD34*	*MYCT1*	*CDH5*	*CXorf36*
*CXorf36*	*CD34*	*RHOJ*	*ELTD1*	*CDH5*	*NOTCH4*	*CXorf36*	*TIE1*
*ELTD1*	*CD93*	*S1PR1*	*ESAM*	*CLEC14A*	*PCDH12*	*TIE1*	
*ERG*	*CDH5*	*SH2D3C*	*RHOJ*	*CXorf36*	*PLVAP*		
*ESAM*	*CLEC14A*	*SHE*	*TIE1*	*DLL4*	*ROBO4*		
*LDB2*	*CXorf36*	*TEK*		*ECSCR*	*S1PR1*		
*MMRN2*	*ELTD1*	*TIE1*		*ELTD1*	*TIE1*		
*MYCT1*	*ERG*			*ERG*			
*TIE1*	*GPR116*			*ESAM*			
	*LDB2*			*GPR4*			

The signatures were defined by an iterative process. Starting with the expanded endothelial gene set (Additional file 10: Table S5) the gene with the lowest mean value of the correlation coefficients of the log2 expression with the genes in the set was removed from the set. The process was reiterated until all genes had a mean correlation coefficient above 0.85

**Fig. 1:**
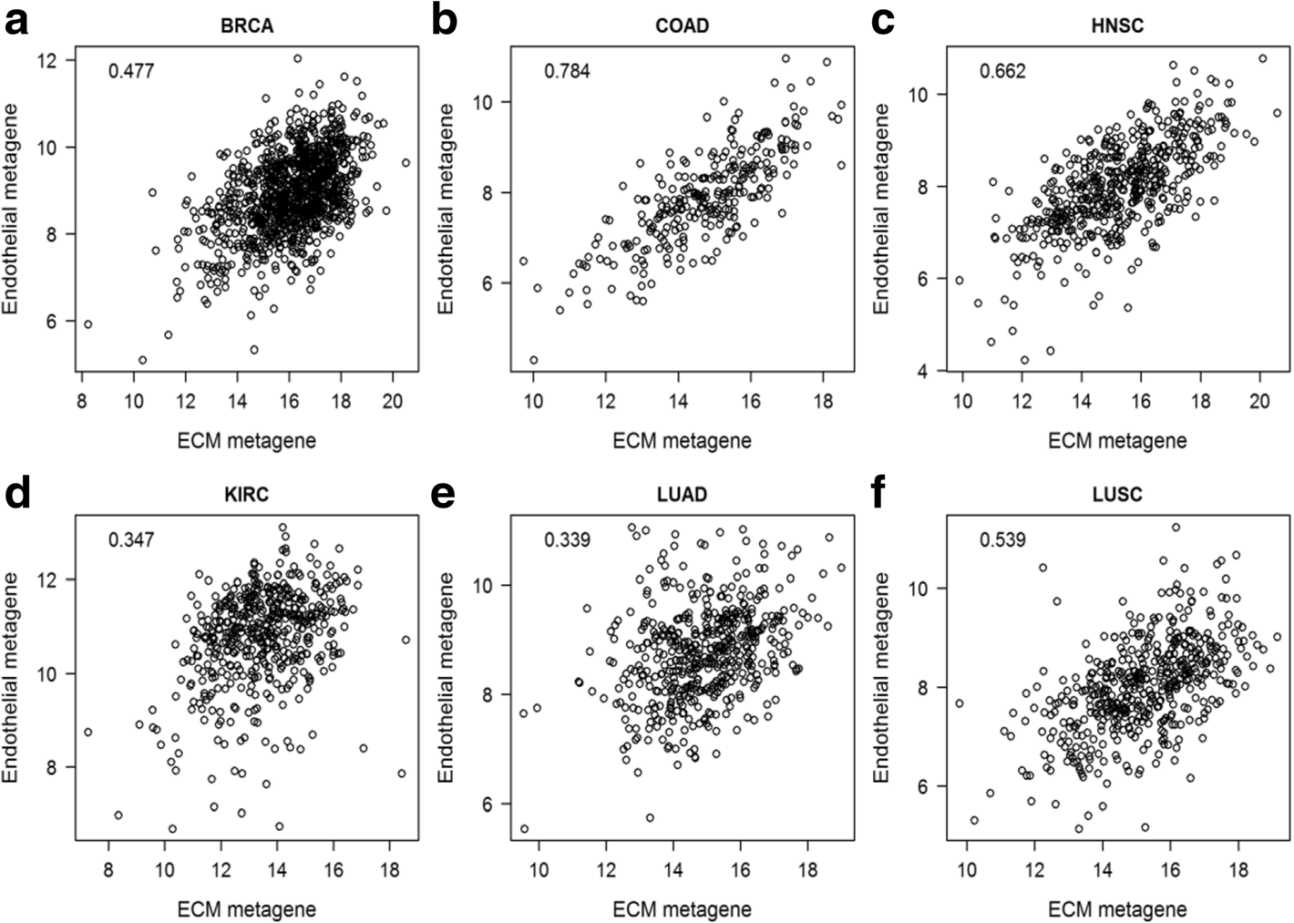
Correlation of ECM and endothelial gene signatures in different cancers. Scatter plots demonstrate mean log2 expression of ECM and endothelial metagenes for individual tumors from the TCGA RNAseq data sets of **a** breast cancer, **b** colon cancer, **c** head and neck cancer, **d** kidney renal clear cell carcinoma, **e** lung adenocarcinoma and **f** lung squamous cell carcinoma. The correlation coefficient is shown in the figure for each data set

To investigate if the ECM and endothelial metagenes are associated with each other, scatter plots were generated with the mean log2 expression level of the metagenes for each tumor as variables (Fig. 1). This revealed a positive correlation between the sets in each tumor form but the strength of the association varies with correlation coefficients ranging from 0.34 in lung adenocarcinoma to 0.78 in colon adenocarcinoma.

### Association of ECM and endothelial signatures with prognosis

There was no association with the magnitude of the metagenes and prognosis in breast cancer, colon cancer and head and neck cancer (Table 3A and B). However, in kidney clear cell carcinoma and lung adenocarcinoma the ECM metagene was associated with poor prognosis and in kidney clear cell carcinoma the endothelial signature with good prognosis following adjustment for the ECM set. For lung squamous cell carcinoma both metagenes were associated with poor prognosis but in a multivariate model only the endothelial signature was significant.

**Table 3:** Cox proportional hazard models for six tumor types using standardized mean values for the gene signature as variables

	Univariate		Multivariate	
	HR	*p*-val	HR	*p*-val
A) ECM				
BRCA	0.961	0.784	1.002	0.991
COAD	0.823	0.387	0.735	0.397
HNSC	0.934	0.697	0.868	0.525
KIRC	1.321	0.014	1.434	0.003
LUAD	1.505	0.005	1.533	0.004
LUSC	1.268	0.047	1.144	0.331
B) Endothelial				
BRCA	0.927	0.564	0.926	0.612
COAD	0.903	0.670	1.170	0.689
HNSC	1.032	0.851	1.122	0.591
KIRC	0.816	0.088	0.729	0.015
LUAD	1.018	0.898	0.915	0.561
LUSC	1.286	0.025	1.202	0.160
C) Breast cancer				
ECM	1.174	0.290		
Endothelial	0.905	0.470		
Estrogen receptor	0.429	<0.001		
Tumor size	2.363	0.014		
Node status	2.120	0.004		

In A and B the signatures were either tested in univariate models or together in multivariate models stratified for age and stage. In C the model also included estrogen receptor status (+/−), tumor size (<2 vs >2 cm), and node status (+/−). All data are from TCGA cancer sets

**Fig. 2:**
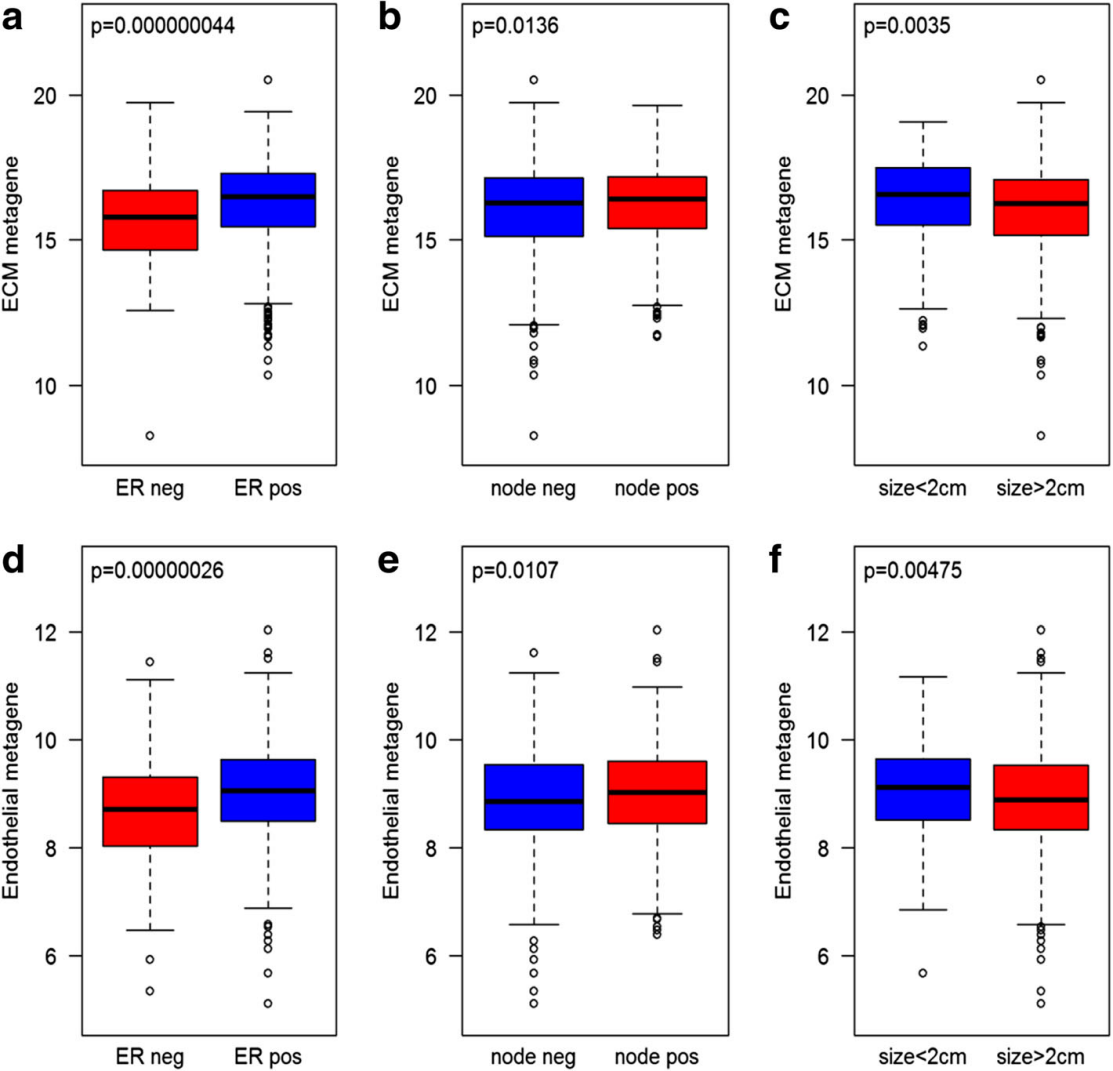
Expression levels of ECM and endothelial metagene in TCGA breast cancer tumors. Graphs demonstrate mean log2 expression levels of genes in the ECM and endothelial gene signatures in individual tumors grouped according to ER status (**a** and **d**), lymph node involvement (**b** and **e**), and tumor size (**c** and **f**)

For breast cancer the association of the metagenes with other prognostic factors was analyzed (Fig. 2). Both metagenes had higher expression values in ER-positive than ER-negative tumors (Fig. 2a, d), suggesting that ER-positive tumors are more stroma and vessel rich which are in line with other studies [24–26]. We also found that smaller tumors and node-positive tumors had slightly higher expression levels of the ECM and endothelial metagenes (Fig. 2b-c, e-f). Since there was an association with the gene signature level and these prognostic markers, a multivariate Cox proportional model for the breast cancer set was evaluated with the metagenes, ER status, node status and tumor size as variables (Table 3C). However, the metagenes were not associated with outcome in this model either.

**Fig. 3:**
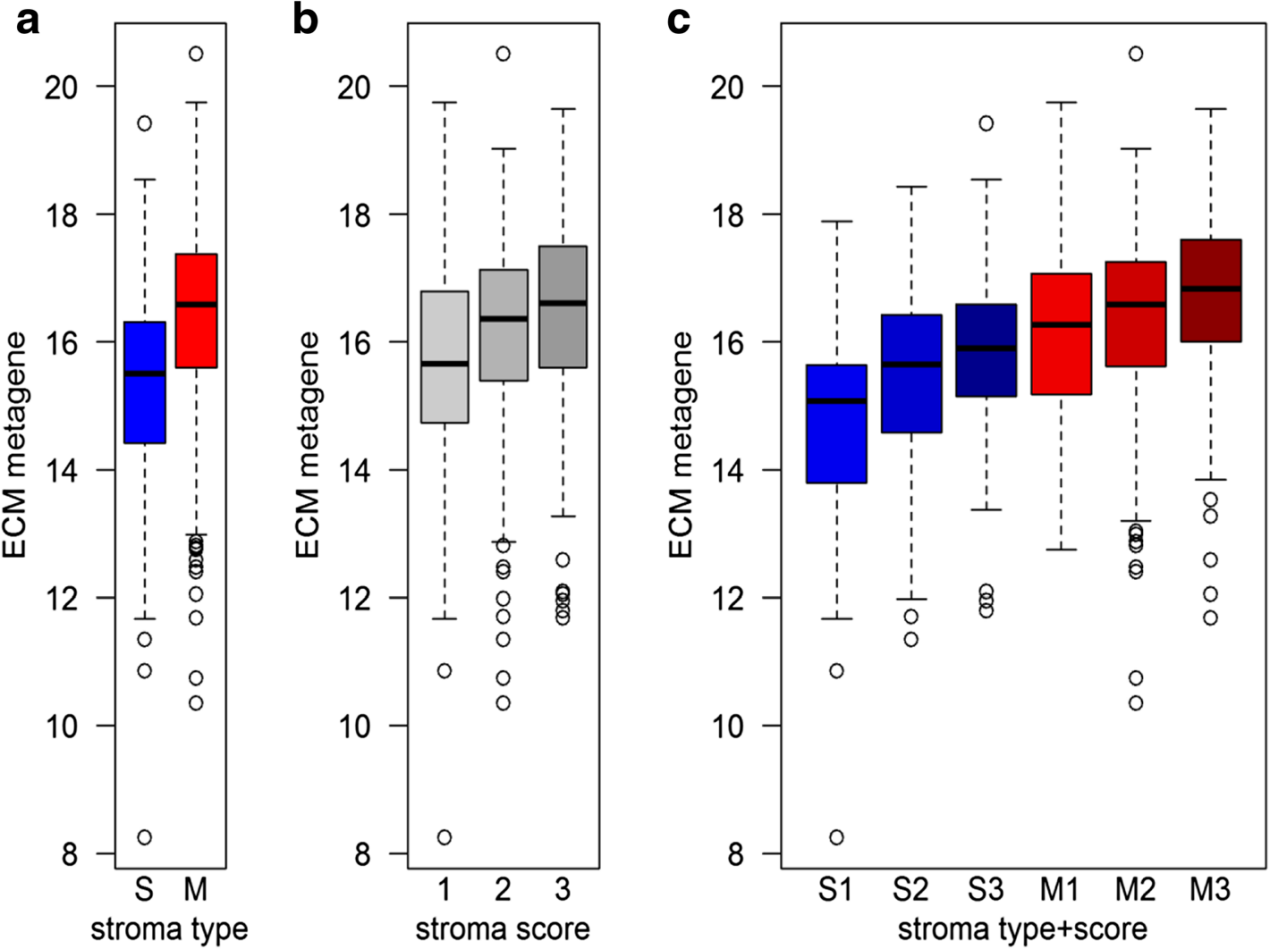
Expression levels of the ECM metagene in breast cancer tumors related to stroma morphology. The mean log2 expression levels of genes in the ECM gene signature are shown for TCGA breast tumors categorized as predominantly having separated (S) or mixed (M) stromal pattern (**a**), or based on a score of the total amount of tumor stroma (**b**). **c** demonstrates the expression level with respect to both stroma type and stroma score

To further delineate what the expression levels of the ECM metagene may represent, the histology slides of the breast cancer TCGA tumors were examined. The tumors were categorized in two groups based on the major pattern of stroma morphology (Additional file 7: Figure S1). The groups represent 1) tumors where the stroma is clearly separated from cancer cells and mainly form belts around nodules of cancer cells and 2) tumors where the stroma is largely mixed with the cancer cells. We found that tumors with the mixed pattern had substantially higher expression levels of the ECM signature (Fig. 3a). A visual quantification of the total amount of stroma was also done and a weak association of the level of the ECM metagene and the amount of stroma was observed (Fig. 3b-c), but the association with stroma type was more evident.

### Prognostic ECM-associated gene sets

**Fig. 4:**
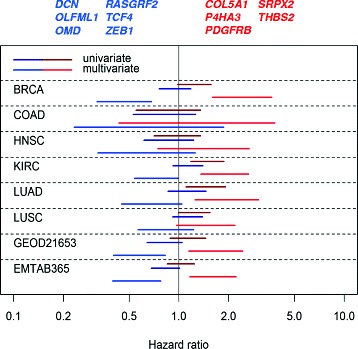
Hazard ratios of gene sets with opposing prognostic association in different cancer data sets. Lines represent confidence intervals (95 %) from multivariate Cox proportional hazard analyses using mean log2 expression of the genes in the prognostic ECM gene signatures (listed on top of the figure) stratifying for age and stage. Red lines and font represent the signature associated with poor prognosis and blue lines and font the signature associated with good prognosis when both signatures are evaluated in a multivariate Cox model. For the TCGA breast cancer (BRCA, which was used for training) and the breast cancer microarray sets (GEOD21653 and EMTAB365) the model was adjusted for node and ER status. The TCGA data sets are colon adenocarcinoma (COAD), head and neck squamous cell carcinoma (HNSC), kidney renal clear cell carcinoma (KIRC), lung adenocarcinoma (LUAD) and lung squamous cell carcinoma (LUSC)

We recently found for immune response genes that compact gene clusters, which are correlated to a substantial degree, in a multivariate Cox model were highly associated with outcome with opposing hazard ratios [17]. However, in univariate models the clusters had no prognostic information. This implies that the molecular balance of the immune response genes, rather than the extent of the immune response, is of importance for prognosis. To investigate if there is a similar phenomenon for ECM-related genes we used the TCGA breast cancer cohort to identify gene pairs that in multivariate models, stratified for node and ER status, had opposing hazard ratios. We thereafter selected the top 100 pairs, based on the *p*-value of the likelihood ratio test of the model. To obtain signatures associated with prognosis, genes that were present in more than five of the top 100 pairs were selected. Depending on if the hazard ratio was >1 or <1 the gene was classified in one of two sets, one associated with good and one with poor prognosis in a multivariate model. The genes in the sets are listed on top of Fig. 4.

The gene sets, quantified as the centered mean log2 expression level of the genes in the set, were evaluated in univariate and multivariate Cox models for prognostic association in several TCGA cancer sets (Fig. 4). Neither set had a consistent association with prognosis in univariate models except for kidney renal clear cell carcinoma and lung adenocarcinoma where the poor prognosis set was coupled to a worse prognosis, perhaps reflecting the association of the ECM score with poor prognosis for these cancer sets.

In the multivariate models the hazard ratios were markedly shifted to larger magnitudes for both gene sets in all TCGA data sets. The most striking effect was seen for the TCGA breast cancer set, which is not surprising since this set was used for training. To validate the breast cancer data the same analysis was done on two breast cancer microarray data sets (Fig. 4). Here the centered mean log2 value of the probe with maximal signal for each gene in the set was used as a measure of the gene set. The Cox model was stratified for ER and node status. Both sets were associated with prognosis in a manner similar to the TCGA cancer sets. Since the genes originally were selected based on the association with stroma this suggests there are characteristics of stroma that are important for prognosis in breast cancer and that to some extent are common for several cancer types.

**Fig. 5:**
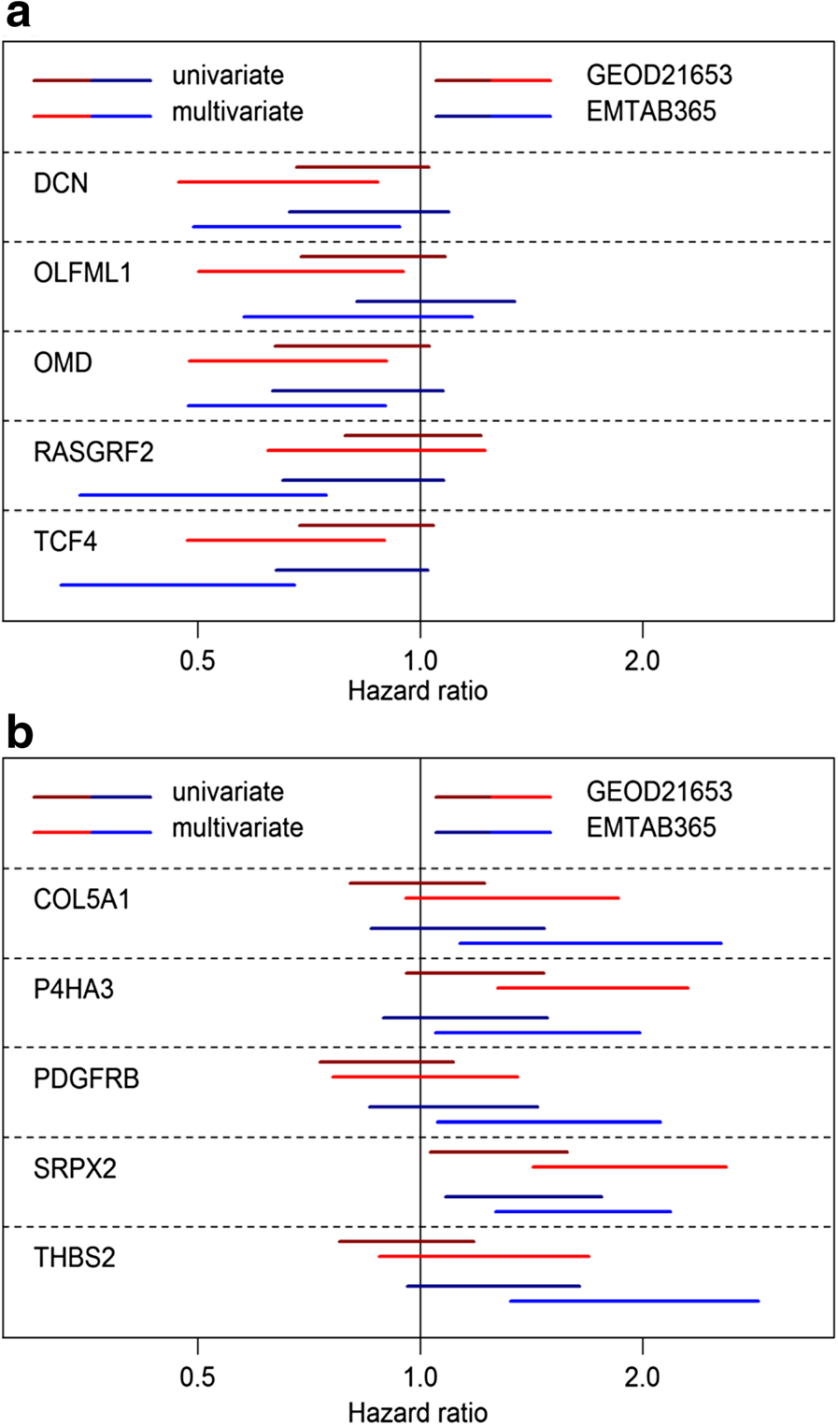
Association of individual genes in the prognostic signatures with prognosis in breast cancer microarray sets. The association with prognosis of the expression of individual genes in the good (**a**) and poor (**b**) prognosis signatures was analyzed using Cox proportional hazards modeling. Figures demonstrate 95 % confidence interval of the hazard ratio for the standardized log2 mRNA expression of indicated genes in a univariate (*dark colors*) or multivariate (*bright colors*) model. In the latter case the aggregated value of the poor prognosis signature (**a**) or good prognosis signature (**b**) was used as additional variable in the model

To further delineate the importance in breast cancer of individual genes in the gene sets, the standardized log2 expression level of each individual gene was used in a multivariate Cox model with the aggregated level of the opposing set as other variable. The analysis was done for the two breast cancer microarray sets (Fig. 5). In univariate models there were few cases when the 95 % confidence interval of the hazard ratio did not include 1, although the genes in the good prognosis set generally had hazard ratios <1 (Fig. 5a) and the genes in the poor prognosis set had ratios >1 (Fig. 5b). When adjusting for the opposing metagene the magnitude of the hazard ratio of essentially all genes was amplified and in many instances it was significant. The 95 % confidence intervals of the hazard ratios of *P4HA3*, *SRPX2, DCN, OMD,* and *TCF4* all excluded 1 in multivariate analysis in both cancer sets. The other mRNAs were significant in one of the microarray sets.

### Expression of prognostic ECM-associated genes

**Fig. 6:**
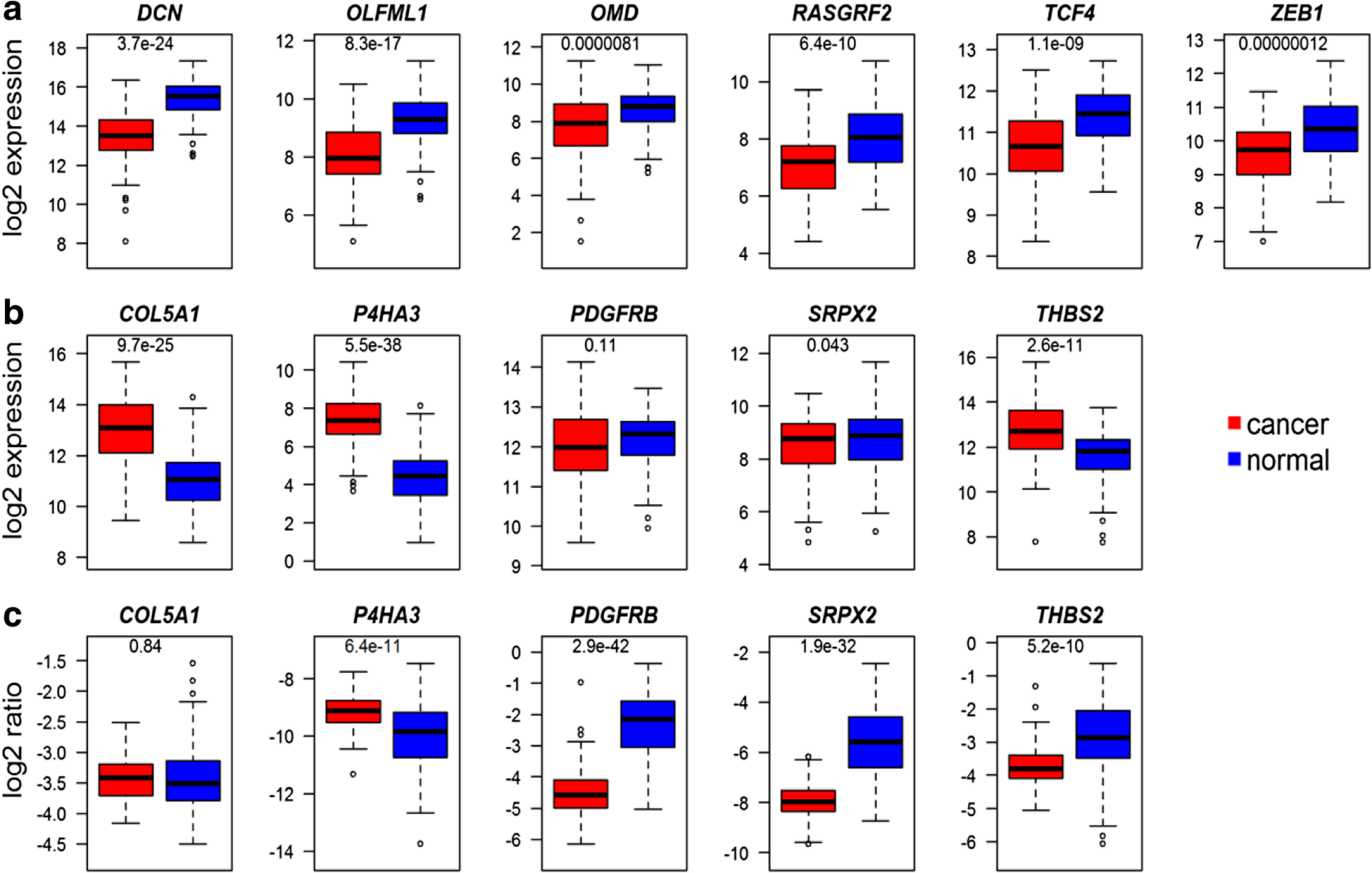
Expression of genes in prognostic signatures in breast cancer versus normal tissue. The mRNA levels of the genes in the good (**a**) and poor (**b**) prognosis signatures in TCGA breast cancer tumors and normal breast tissue from the same patient. Since the ECM metagene is expressed at higher levels in cancer tissue the levels of the genes in (**b**) were normalized to the mean log2 value of the ECM metagene (**c**). The *p*-value of a *t*-test comparing the two groups is indicated in each figure

To further analyze these genes their expression in breast cancer tissue and accompanying normal tissue from the TCGA data set was assessed (Fig. 6). For the genes in the signature associated with improved prognosis all genes were expressed at higher levels in normal breast tissue (Fig. 6a), indicating that their down regulation may be coupled to malignancy. For the genes associated with poor prognosis three out of five were expressed at lower levels in normal tissue. However, the interpretation of this result is complicated by the fact that the ECM metagene also is lower in normal tissue. Thus, the lower expression of these genes may be a consequence of fewer ECM-producing cells. Their expression was therefore normalized to the ECM metagene (Fig. 6c). Following this adjustment only *P4HA3* was higher in cancer tissue.

**Fig. 7:**
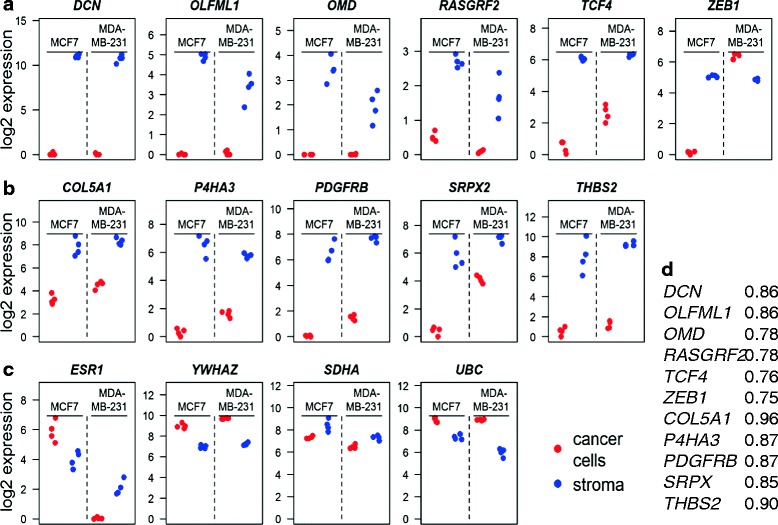
Expression of prognostic signature genes in tumor stroma. The mRNA expression data from the xenografted tumors in [22] were downloaded. The RPKM data of a gene was divided by the total sum of RPKM in the sample and multiplied by 100,000. The normalized value was log2 transformed following addition of 1. The resulting values for the genes in the good (**a**) or poor (**b**) prognosis in each tumor are depicted. As a comparison, values of selected house-keeping genes or cancer-related genes are shown in (**c**). The correlation coefficients for each gene in the prognostic signatures with the ECM metagene in TCGA breast cancers are shown in (**d**)

Our hypothesis was that all genes investigated are primarily stromal and expressed in matrix-producing cells. To further investigate this assumption we utilized data from an elegant study where mRNAs from cancer cells and stroma can be separated based on species differences using human breast cancer cell lines grafted into mice [22]. In all samples of grafts of either MDA-MB-231 or MCF-7 cells, all genes in both prognostic sets were markedly higher in the stroma (Fig. 7a-b). The only exception was *ZEB1* which was at similar levels in the cancer cells and in the stroma in the MDA-MB-231 tumors. Furthermore, all the mRNAs in the two prognostic sets also had a high correlation coefficient with the ECM metagene in the TCGA breast cancers further supporting their expression in stroma (Fig. 7d).

**Fig. 8:**
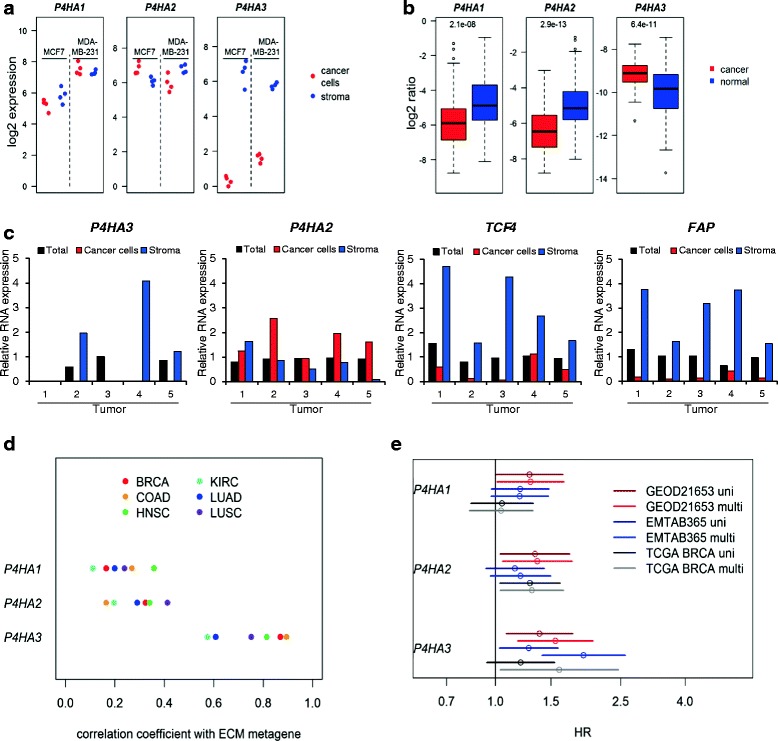
Expression and prognostic association of *P4HA* gene mRNAs. The expression of *P4HA1* and *P4HA2* was calculated in the stroma and cancer cells using the xenograft data (**a**) and in breast cancer and normal breast tissue normalized to the ECM metagene using the TCGA data (**b**). The *P4HA3* data, also shown in Figs. 6 and 7 are included for comparison. Five breast cancer tumors were laser-microdissected and the mRNA levels of indicated genes were analyzed in the cancer cell and stroma compartments (**c**). The correlation coefficients of *P4HA1*, *P4HA2*, and *P4HA3* with the ECM metagene in six different TCGA cancer sets were calculated (**d**). The hazard ratios of the standardized log2 mRNA expression of the *P4HA* genes were estimated using Cox proportional hazards model (**e**). The lines indicate 95 % confidence intervals of the hazard ratios in univariate models (*dark colors*) or multivariate models (*bright colors*) in which the standardized ECM metagene was included as variable. All models were stratified for node and ER status

*P4HA3* was the only mRNA that was expressed at higher levels in cancer compared to normal breast tissue following adjustment for the ECM metagene and it was one of the genes that were significantly associated with prognosis in the multivariate models in both microarray sets. P4HA3 is a collagen-modifying enzyme causing 4-hydroxylation of prolines [27]. The reaction is also catalyzed by the closely related proteins P4HA1 and P4HA2. The hydroxylation is a prerequisite for efficient collagen I triple helix formation. These genes were therefore compared regarding stromal enrichment and association with prognosis. In the xenografted tumors both *P4HA1* and *P4HA2* mRNA are found at similar levels in cancer cells and in stroma, contrasting the stroma-specific expression of *P4HA3* (Fig. 8a). Furthermore, neither ECM metagene-adjusted *P4HA1* nor *P4HA2* levels were higher in breast cancer than in normal tissue, which also contrasts what was found for *P4HA3* (Fig. 8b).

To further substantiate the stroma-specific expression of *P4HA3* we microdissected five breast cancer tumors and analyzed the mRNA in the stroma and cancer cell compartments (Fig. 8c). In the tumor *P4HA3* mRNA was detected in the stroma compartment in three out of five tumors and it was undetectable in the cancer cell compartment of all tumors. On the other hand, *P4HA2* mRNA was detected at higher levels in the cancer cell compartment of all tumors but one. We also analyzed *TCF4*, which was found in the positive prognosis set, and *FAP*, a marker for cancer associated fibroblasts. Both genes were enriched in the stromal compartment of all tumors investigated.

To analyze whether the stroma association of the *P4HA* genes is a feature common across cancer types the correlation coefficients of the *P4HA* mRNAs with the ECM metagene was analyzed in six TCGA sets (Fig. 8d). In all cancers *P4HA3* was the gene whose expression had the highest correlation coefficient with the ECM metagene.

In addition, we assessed the association of the expression levels of *P4HA* genes with prognosis using the TCGA and the two microarray breast cancer sets (Fig. 8e). The *P4HA* mRNAs were to a varying extent associated with poor prognosis in all sets. However, when adjusting for the ECM metagene by including it in a multivariate model, the magnitude of the hazard ration was enhanced only for *P4HA3*.

## Discussion

In this study we have taken an approach to utilize RNA-seq data to obtain an estimation of the amount of stroma and vascularization in a tumor and to analyze if these estimates or the mRNA composition of the stroma are associated with prognosis. Through this approach we have identified two gene sets, which in breast cancer when adjusted for each other are associated with poor and good prognosis, respectively. This highlights potential genes that may determine the contribution to malignancy of the tumor stroma.

A tumor consists of a wide range of cell types. It is conceivable that in many instances cells of the same type have a set of genes that are fairly specific for the type. Unless they are differentially regulated the expression of such genes would be expected to be highly correlated and related to the amount of the cells in the specimen. This reasoning was the basis for the search for compact clusters of genes using the mean correlation coefficient of the log2 expression as a similarity measure. For the ECM-related genes the size of the cluster varied with tumor type. The common genes were all collagens indicating that it represents the amount of collagen-producing cells such as fibroblasts or other mesenchymal cells. This is further supported by other genes that correlated closely in many cancer forms such as *FAP*, *PDGFRB*, *THBS2* and *VCAN* which are also markers for matrix-producing cells and in some cases also for cancer-associated fibroblasts [28–30].

The largest ECM cluster was obtained for the colorectal adenocarcinoma data set whereas the smallest cluster was seen for kidney renal clear cell carcinoma. This could be due to a higher stroma fraction in colorectal adenocarcinomas which would conceivably result in higher signal to noise ratios for the stromal genes and consequently larger correlation coefficients. In fact, the renal clear cell carcinomas had the lowest expression level of the ECM metagene (Additional file 11: Figure S3A). On the other hand, this cancer form had the highest expression level of the endothelial metagene (Additional file 11: Figure S3B), and here the compact cluster, together with colorectal adenocarcinoma, contained the largest number of genes among the cancer types. Thus, there may be a relation between the expression level of the metagene and the number of genes that will be found in a compact cluster. Kidney renal clear cell carcinomas are frequently highly vascularized due to deletion or inactivation of the VHL suppressor gene which results in upregulation of hypoxia-inducible factors and angiogenic factors such as VEGF [31]. Our data are in line with this.

To obtain general signatures for the stromal components the final metagenes were based on the clusters obtained from six separate cancer forms. This will conceivably increase the likelihood for obtaining metagenes stable over several cancer forms. The requirement reduced the number of genes in the ECM and endothelial signatures to three in each set. The levels of the ECM and endothelial metagenes correlated positively in all cancer forms to varying extent. Thus, there may be interdependency between the ECM production and the amount of endothelial cells in a tumor or one may be influenced by the other. It has for instance been shown that ECM-producing fibroblasts promote endothelial lumen formation [32].

The tumor specimen taken for RNA analysis may contain surrounding non-tumor tissue. This could potentially influence both the extent and type of mRNA derived from stroma and thereby the interpretations of for instance survival analyses and correlations with clinicopathological parameters of the metagenes. However, there was an association of the ECM metagene with the stroma pattern in the tumor (Fig. 3) suggesting that at least the ECM metagene to some extent represents features of the intratumor stroma. Using the mean log2 expression of these genes as indicator for the amount of ECM-producing cells and endothelial cells no association with prognosis could be detected in most cancer types analyzed. However, for both renal clear cell carcinoma and lung adenocarcinoma higher levels of the ECM score was associated with worse prognosis. Furthermore, when adjusting for the ECM signature, the endothelial set was associated with an improved prognosis in renal clear cell carcinoma. Thus, for a given level of stroma-producing cells an increased vascularization may indicate a better prognosis for kidney renal clear cell carcinoma.

For breast cancer no association for the ECM metagene with prognosis was observed, neither in univariate nor multivariate models when adjusting for other prognostic factors. This is analogous to what we have also seen for immune-related sets [17]. However, as for the immune genes it was possible to identify ECM gene sets that when evaluated in a multivariate Cox model had opposite associations with prognosis. The sets had a similar association with prognosis in multivariate models in several cancer forms. In two breast cancer microarray sets the pattern was the same as in the TCGA breast cancer set with the two gene signatures being not so strongly associated with prognosis in univariate Cox models but being clearly associated with prognosis in the multivariate setting. Thus, the molecular composition of the stroma-producing cells may be more important for breast cancer prognosis than the extent of stroma production in the tumor.

In terms of individual genes in the prognostic signatures *TCF4* was perhaps the most striking example in the set associated with improved prognosis. Its association with prognosis was not so strong in a univariate model but when adjusting for the poor prognosis set it had a clear association with improved prognosis. This may be somewhat counterintuitive given the vast number of reports that have linked β-catenin/TCF with oncogenic effects following the discovery of Tcf as a component in the mediation of malignancy induced by APC deletion [33]. However, the data shown here represented by the high correlation coefficient between *TCF4* and the ECM metagene, the higher expression of *TCF4* in the stroma of both xenografted tumors and laser-microdissected breast cancer tumors indicate that in breast cancer the bulk of *TCF4* mRNA emanates from matrix-producing cells. Here its expression may actually oppose a malignant development of the tumor given its association with improved prognosis. Such an assumption is supported by the fact that *TCF4* is found at higher levels in normal than in breast cancer tissue in the TCGA set and in another material [34].

For the genes in the poor prognosis set *P4HA3* was associated with poor prognosis in the multivariate setting in both microarray sets. It was also the only mRNA in the poor prognosis signature that after normalization to the ECM metagene was expressed at higher levels in cancer than in normal tissue. The P4HA proteins catalyze a 4-hydroxylation on prolines in collagen. P4HA1 and P4HA2 in the cancer cells have been shown to be critical for breast cancer cell line collagen deposition, invasion and metastasization [35]. They are also induced in fibroblasts by hypoxia and there contributes to production of a matrix that promotes breast cancer cell migration [36]. The P4HA-mediated reaction takes place intracellularly and it would therefore be expected that the magnitude of *P4HA* gene expression would primarily correlate with collagen gene expression. However, this is only the case for *P4HA3. P4HA3* is the only *P4HA* gene that seems to be selectively expressed in the stroma of tumors. *P4HA3* is also the only *P4HA* gene whose association with poor prognosis in a Cox model is enhanced upon adjustment for an ECM metagene. This highlights *P4HA3* expression as a marker for malignant stroma and for stroma that contributes to poor prognosis. It is in this context of interest to note that P4HA3 has been identified as an important contributor to TGFβ-mediated pulmonary fibrosis [37]. Our data indicate an additional role for P4HA3 in the matrix-producing cells in the tumor stroma.

## Conclusions

In conclusion this study indicates that the amount of stroma or endothelial tissue is not a strong prognostic factor in breast cancer. However, the molecular balance in the matrix-producing cells is associated with prognosis. Here we provide indications that the expression levels of *TCF4* and *P4HA3* in the stroma are associated with prognosis. They may therefore contribute to (*P4HA3*) or suppress (*TCF4*) the malignant contributions of stroma.

## Additional files

Additional file 1: Table S1. TCGA data sets. (PDF 25 kb)
Additional file 2: Table S2. Breast cancer data sets. (PDF 26 kb)
Additional file 3: Table S3. Expanded ECM gene set used for identification of compact ECM cluster. (PDF 30 kb)
Additional file 4: The R code used to generate signatures, figures and tables. (R 31 kb)
Additional file 5: R object containing signatures defined in reference [17]. (RDATA 704 bytes)
Additional file 6: Information in Additional file 10 in Excel format. (XLSX 10 kb)
Additional file 7: Figure S1. Histological analysis of TCGA breast tumors. Tumors with distinct and compactly organized stroma surrounding a bulk tumorous structure and were classified as tumors with “separated” stroma (A) whereas tumors with a more integrated pattern of stroma and cancer cells were classified as “mixed” (B). (PDF 256 kb)
Additional file 8: Figure S2. Laser Microdissection. Representative pictures of a cresyl violet stained triple-negative breast tumor (A), marked (B) and dissected (C) for inflammatory stromal compartment. (PDF 164 kb)
Additional file 9: Table S4. ID of TaqMan® gene expression assays. (PDF 41 kb)
Additional file 10: Table S5. Expanded endothelial gene set used for identification of compact endothelial cluster. (PDF 28 kb)
Additional file 11: Figure S3. Levels of ECM and endothelial metagenes in different cancer types. The expression levels of the ECM (A) and endothelial (B) metagenes were quantified in TCGA RNA-seq cancer sets (BRCA – breast adenocarcinoma, COAD – colon adenocarcinoma, HNSC – head and neck squamous cell carcinoma, KIRC – kidney renal clear cell carcinoma, LUAD – lung adenocarcinoma, LUSC – lung squamous cell carcinoma). (PDF 121 kb)

## Abbreviations

ECM: Extra cellular matrix
LMD: Laser microdissection
TCGA: The cancer genome atlas
