# Supplementary material for: The expression pattern of matrix-producing tumor stroma is of prognostic importance in breast cancer
Source: BMC Cancer. 2016 Nov 4;16:841. doi: 10.1186/s12885-016-2864-2 (PMC5095990; doi:10.1186/s12885-016-2864-2)
Supplement: Additional file 1: Table S1. — TCGA data sets. (PDF 25 kb) [file 12885_2016_2864_MOESM1_ESM.pdf]

Table S1. TCGA data sets.

TCGA set	Abbreviation	**mRNA data download** (date YYMMDD)	**Survival data download** (date YYMMDD)	End point
Breast Carcinoma	BRCA	150130	150918	NTE
Colon Adeno-carcinoma	COAD	140806	150917	OS
Head and Neck Squamous Cell Carcinoma	HNSC	140806	150918	OS
Kidney Renal Clear Cell Carcinoma	KIRC	140115	150918	OS
Lung Adeno-carcinoma	LUAD	140806	150917	OS
Lung Squamous Cell Carcinoma	LUSC	140806	150917	OS

OS – Overall survival, NTE – New tumor event
