# Supplementary material for: The expression pattern of matrix-producing tumor stroma is of prognostic importance in breast cancer
Source: BMC Cancer. 2016 Nov 4;16:841. doi: 10.1186/s12885-016-2864-2 (PMC5095990; doi:10.1186/s12885-016-2864-2)
Supplement: Additional file 2: Table S2. — Breast cancer data sets. (PDF 26 kb) [file 12885_2016_2864_MOESM2_ESM.pdf]

Table S2. Breast cancer data sets.

**Dataset**	**Source**	**Download** (date YYMMDD)	**No of samples**	**ER** (neg/pos)	**Node status** (neg/pos)	**Endpoint**	**Event** (no/yes)	**Median followup time** (days)
TCGA Breast Cancer	TCGA data portal	150130	1086	226/779	597/536	NTE	763/110	858
GEOD21653	ArrayExpress	140811	266	113/150	120/140	DFS	169/83	1609
EMTAB365	ArrayExpress	140805	537	108/407	139/299	MFS	408/119	1886

NTE – New tumor events, DFS – Disease-free survival, MFS –metastasis-free survival
