# Supplementary material for: The expression pattern of matrix-producing tumor stroma is of prognostic importance in breast cancer
Source: BMC Cancer. 2016 Nov 4;16:841. doi: 10.1186/s12885-016-2864-2 (PMC5095990; doi:10.1186/s12885-016-2864-2)
Supplement: Additional file 3: Table S3. — Expanded ECM gene set used for identification of compact ECM cluster. (PDF 30 kb) [file 12885_2016_2864_MOESM3_ESM.pdf]

Table S3. Expanded ECM gene set used for identification of compact ECM cluster

ADAM12	*DACT1*	*NOX4*
ADAMTS12	*DCN*	*OLFML1*
ADAMTS2	*ECM2*	*OLFML2B*
AEBP1	*FAM26E*	*OMD*
ANTXR1	*FAP*	*P4HA3*
ASPN	*FBN1*	*PCOLCE*
BNC2	*FIBIN*	*PDGFRB*
C10orf72	*FN1*	*PODN*
C14orf37	*FSTL1*	*POSTN*
CCDC80	*GLT8D2*	*PRRX1*
CDH11	*GXYLT2*	*RASGRF2*
CHSY3	*HEG1*	*RECK*
COL10A1	*HMCN1*	*SERPINF1*
COL12A1	*HTRA1*	*SFRP2*
COL1A1	*ITGA11*	*SPARC*
COL1A2	*KIAA1462*	*SRPX2*
COL3A1	*LOX*	*TCF4*
COL5A1	*LRRC15*	*THBS2*
COL5A2	*LUM*	*THY1*
COL6A1	*MAGEL2*	*TIMP2*
COL6A2	*MFAP5*	*TSHZ3*
COL6A3	*MMP2*	*VCAN*
COL8A1	*MSRB3*	*WISP1*
COL8A2	*MXRA5*	*ZEB1*
CTSK	*NID1*	*ZFPM2*
CYS1	*NID2*	
