# Supplementary material for: The expression pattern of matrix-producing tumor stroma is of prognostic importance in breast cancer
Source: BMC Cancer. 2016 Nov 4;16:841. doi: 10.1186/s12885-016-2864-2 (PMC5095990; doi:10.1186/s12885-016-2864-2)
Supplement: Additional file 7: Figure S1. — Histological analysis of TCGA breast tumors. Tumors with distinct and compactly organized stroma surrounding a bulk tumorous structure and were classified as tumors with “separated” stroma (A) whereas tumors with a more integrated pattern of stroma and cancer cells were classified as “mixed” (B). (PDF 256 kb) [file 12885_2016_2864_MOESM7_ESM.pdf]

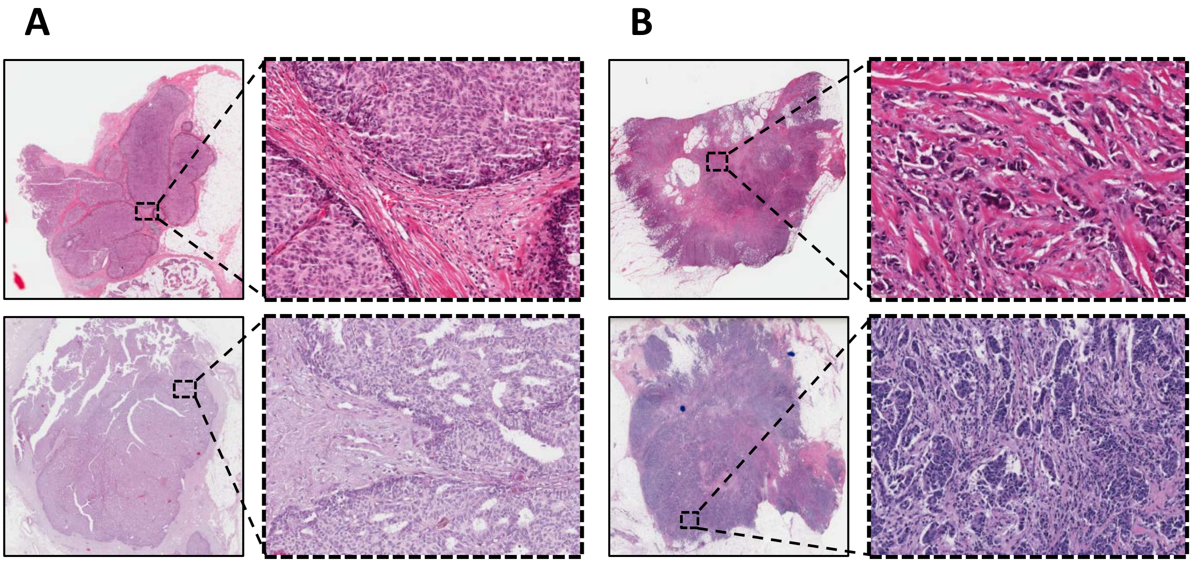

Supplementary Figure 1. Histological analysis of TCGA breast tumors. Tumors with distinct structure and compactly organized stoma surrounding a bulk tumorous structure were classified as tumors with ”separated” stroma (A) whereas tumors with a more integrated pattern of stroma and cancer cells were classified as ”mixed” (B).
