# Supplementary material for: The expression pattern of matrix-producing tumor stroma is of prognostic importance in breast cancer
Source: BMC Cancer. 2016 Nov 4;16:841. doi: 10.1186/s12885-016-2864-2 (PMC5095990; doi:10.1186/s12885-016-2864-2)
Supplement: Additional file 8: Figure S2. — Laser Microdissection. Representative pictures of a cresyl violet stained triple-negative breast tumor (A), marked (B) and dissected (C) for inflammatory stromal compartment. (PDF 164 kb) [file 12885_2016_2864_MOESM8_ESM.pdf]

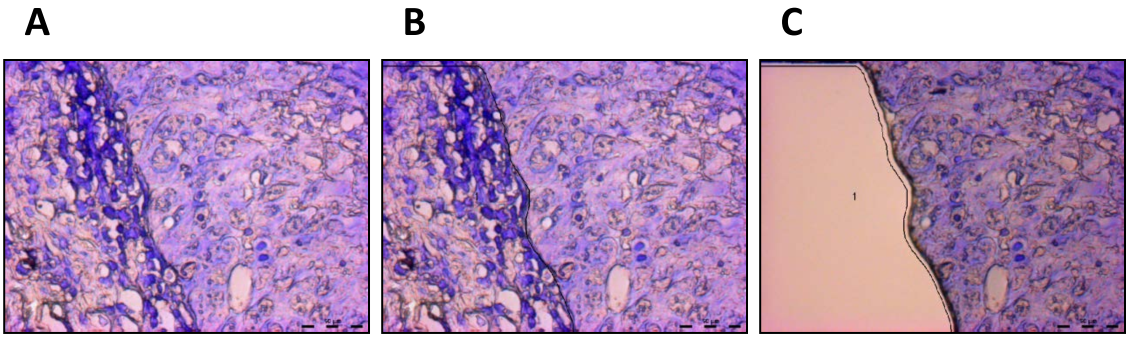

Supplementary Figure 2. Laser Capture Microdissection. Representative pictures of a cresyl violet stained triple-negative breast tumor (A), marked (B) and dissected (C) for inflammatory stromal compartment.
