# Supplementary material for: The expression pattern of matrix-producing tumor stroma is of prognostic importance in breast cancer
Source: BMC Cancer. 2016 Nov 4;16:841. doi: 10.1186/s12885-016-2864-2 (PMC5095990; doi:10.1186/s12885-016-2864-2)
Supplement: Additional file 9: Table S4. — ID of TaqMan® gene expression assays. (PDF 41 kb) [file 12885_2016_2864_MOESM9_ESM.pdf]

Table S4. IDs of TaqMan® gene expression assays

Gene name	Assay ID
FAP	Hs00990806_m1
TCF4	Hs00162613_m1
P4HA2	Hs00990001_m1
P4HA3	Hs00420085_m1
ACTB	Hs01060665_g1
UBC	Hs01871556_s1
