# Supplementary material for: The expression pattern of matrix-producing tumor stroma is of prognostic importance in breast cancer
Source: BMC Cancer. 2016 Nov 4;16:841. doi: 10.1186/s12885-016-2864-2 (PMC5095990; doi:10.1186/s12885-016-2864-2)
Supplement: Additional file 10: Table S5. — Expanded endothelial gene set used for identification of compact endothelial cluster. (PDF 28 kb) [file 12885_2016_2864_MOESM10_ESM.pdf]

Table S5. Expanded endothelial gene set used for identification of compact endothelial cluster

ACVRL1	*ERG*	*PREX2*
ADCY4	*ESAM*	*PTPRB*
ARHGEF15	*GJA4*	*RHOJ*
BCL6B	*GNG11*	*ROBO4*
CALCRL	*GPR116*	*S1PR1*
CD34	*GPR4*	*SH2D3C*
CD93	*LDB2*	*SHE*
CDH5	*LHFP*	*SOX17*
CLDN5	*LRRC70*	*TEK*
CLEC14A	*MMRN2*	*THSD1*
CXorf36	*MYCT1*	*TIE1*
DLL4	*NOTCH4*	*USHBP1*
ECSCR	*PCDH12*	*VWF*
ELTD1	*PDE2A*	
EMCN	*PLVAP*	
