# Supplementary material for: The expression pattern of matrix-producing tumor stroma is of prognostic importance in breast cancer
Source: BMC Cancer. 2016 Nov 4;16:841. doi: 10.1186/s12885-016-2864-2 (PMC5095990; doi:10.1186/s12885-016-2864-2)
Supplement: Additional file 11: Figure S3. — Levels of ECM and endothelial metagenes in different cancer types. The expression levels of the ECM (A) and endothelial (B) metagenes were quantified in TCGA RNA-seq cancer sets (BRCA – breast adenocarcinoma, COAD – colon adenocarcinoma, HNSC – head and neck squamous cell carcinoma, KIRC – kidney renal clear cell carcinoma, LUAD – lung adenocarcinoma, LUSC – lung squamous cell carcinoma). (PDF 121 kb) [file 12885_2016_2864_MOESM11_ESM.pdf]

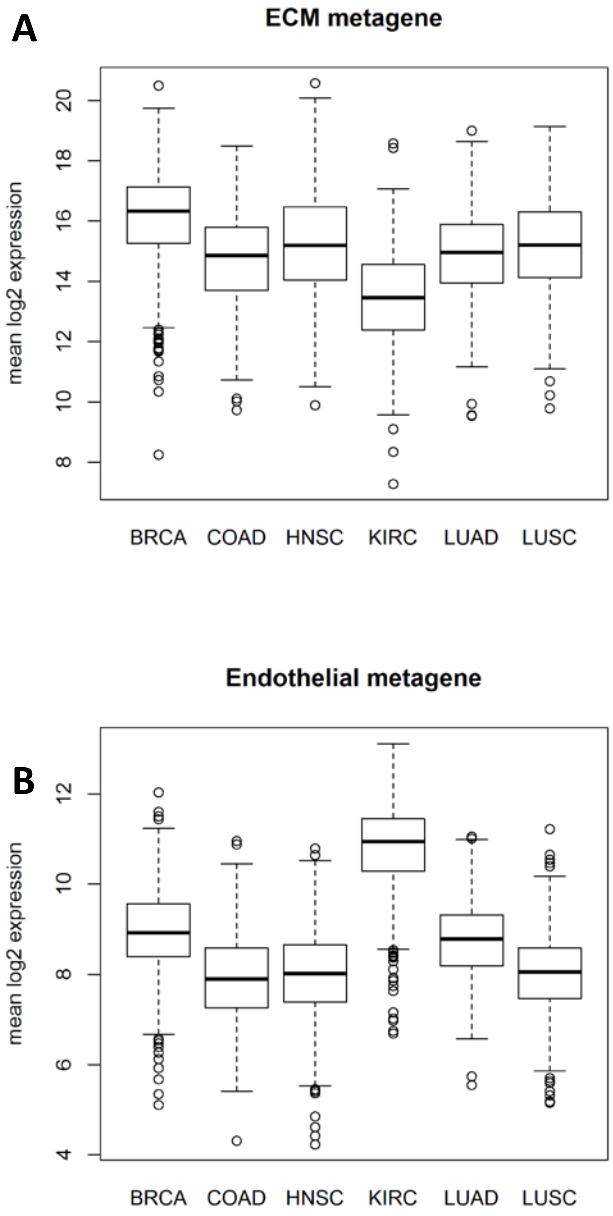

Supplementary Figure 3. Levels of ECM and endothelial metagenes in different cancer types. The expression levels of the ECM (A) and endothelial (B) metagenes were quantified in TCGA RNAseq cancer sets (BRCA – breast adenocarcinoma, COAD – colon adenocarcinoma, HNSC – head and neck squamous cell carcinoma, KIRC – kidney renal clear cell carcinoma, LUAD – lung adenocarcinoma, LUSC – lung squamous cell carcinoma)
